# Research trends of sustainability and marketing research, 2010–2020: Topic modeling analysis

**DOI:** 10.1016/j.heliyon.2023.e14208

**Published:** 2023-03-05

**Authors:** Yeo Jin Jung, Youngmin Kim

**Affiliations:** a Center for Entrepreneurship Studies, Dong-A University, 225 Gudeok-ro, Seo-gu, Busan, Republic of Korea; b Da Vinci College of General Education, Chung-Ang University, 84 Heukseok-ro, Dongjak-gu, Seoul, Republic of Korea

**Keywords:** Sustainability, Marketing, Topic modeling, Latent dirichlet allocation (LDA), Research trend

## Abstract

In recent decades, rapid growth has been observed in the incorporation of sustainability into marketing. Accordingly, the contrasting relationships between them have been carefully studied to assess the relevance of one idea to the other and vice versa. In response to this change, scholars and practitioners have been tasked with exploring the trends in sustainability and marketing. Therefore, the purpose of this study is to investigate existing literatures on both sustainability and all levels of marketing, determine the research trends and provide implications of applying the trends for future research and practices. This research has investigated only the title, abstract, and keywords of 2147 articles that were published between 2010 and 2020 in SSCI or SCIE indexed journals by applying the topic modeling based on the Latent Dirichlet Allocation (LDA) model. The results show that the research trend has shifted from general sustainable concept to more environmental and industrial technology based on the empirical evidence of 14 latent topics of sustainability and marketing. This article aids in understanding the recent research trend in sustainability and marketing, and the findings will be a valuable resource for future scholars and practitioners. It contributes to both existing and future literatures by providing valuable insights from recent research trend in sustainability and marketing and by providing recommendations for future research avenue. Among other bibliometric review articles, this is the most up-to-date comprehensive and empirical article, providing overview of the research trend.

## Highlights

• 14 research topics of sustainability and marketing over 2010–2020 were obtained from the Latent Dirichlet Allocation.
• Two hot topics and one cold topic were identified.
• The research trend has shifted from general sustainability concept to environmental and industrial technology.

## 1 Introduction

Incorporation of sustainability in marketing research has rapidly grown over the last two decades. According to the McKinsey survey, companies have been actively integrating sustainability principles into their businesses to contribute to society and the environment [[1], [2], [3], [4]]. This sustainability dominant perspective of businesses specifically emphasizes economic growth concerning market and consumption and technological solutions for environmental and social problems [[4], [5], [6]]. Moreover, following the COVID-19 pandemic, sustainability has become a topic of discussion worldwide, and several companies and organizations have proposed sustainable frameworks for both businesses and societies as a response to serious environmental, societal, and financial issues [[6], [7], [8], [9], [10], [11]]. In such periods of anxious turbulence, marketing scholars and practitioners recognize that sustainability is a well-known matter in every business sector [1,12,13]. Thus, modern companies are bound to make systemic changes and, subsequently, embrace sustainable approaches that are contingent on dynamic market conditions [14,15]. For marketing scholars and practitioners, this area of research provides opportunities to investigate sustainability issues related to climate change, energy consumption, advertising, consumer behaviors, communication, branding, business practices, environmental concerns, marketing ethics, innovation, and micromarketing [6,11,13,14,[16], [17], [18], [19]]. There has been a consequential growth in “green,” “social,” “environment,” “sustainable,” and “ESG (environment, society, and governance)” marketing, which has focused on promoting “environmentally friendly” products, understanding industries and market segments, and the role of the environment in the industries [4,6,15,20,21].

Sustainability has been on the list of global megatrends since the concept first appeared at the United Nations (UN) conference in 1972, where numerous disciplines raised ethical issues [9,16,[22], [23], [24], [25]], where numerous from various disciplines raised ethical issues [6,26]. It has become an imperative research topic with varying interpretations and contrasting viewpoints and is generally associated with a positive moral standing in social understanding and academic discourse [6,22,[26], [27], [28], [29]]. Although some scholars argue that the UN's concept of sustainability goals is not feasible, scholars and practitioners have recognized the significant role of sustainability as an integrated research field and as an integral component of business strategy [6,30]. Marketing growth has witnessed the concurrence [7,8,[31], [32], [33]] that the future of business depends on the fundamental and consequential compliance with sustainability. The term “sustainability” is employed extensively across diverse disciplines including engineering, social science, liberal arts, science, and various business sectors [11,15,29,[34], [35], [36], [37]]. It provides a long–term future perception focusing on ethical values and principles, which guides harmonious and responsible actions for incorporating environmental, societal, and economic goals [16,17,22]. Each discipline defines sustainability based on its contribution to the common goal of a sustainable future. Sustainability has merged with marketing and extends to executive and social issues [3,37]. It pursues a compound approach and adaption to the varying need for sustainability from both business and academic standpoints in the absence of an appropriate and objective analysis of the interface between sustainability and marketing.

Sustainability has become a significant component of business strategies; however, marketing and sustainability are opposing ideas. Specifically, marketing concerns selling more products and is generally associated with competitive business strategies, profit, and the promotion of consumers' choices that provide instant satisfaction and self–gratification [6,22,27,29]. However, sustainability requires lower product consumption [28,31,33,39]. Previous studies have focused on identifying market segments that have pro-sustainability values, beliefs, and behavioral intentions, including the paradox between consumers’ preferences, attitudes, and intentions and their actual behaviors, by applying innovative psychological and sociological techniques to improve research quality [[38], [39], [40]]. Conversely, marketers consider designing products that are more sustainable, which consumers are willing to purchase [14,22]. Previous studies have aimed to normalize the consumption of sustainable featured products through a better understanding of market needs and marketing skills [27,41,42]. This approach is driven by customer-related sustainable product design, in which sustainable marketing focuses on the producer rather than the market [43].

According to stakeholder theory [44], companies must regard all stakeholder demands to validate their business activities, such as the disclosure of any information related to the profit and value of companies [25,[45], [46], [47], [48]]. Stakeholder theory contends that companies must operate an interconnected network of stakeholders while achieving sustainable profit maximization [44,47,48]. Furthermore, satisfying stakeholders would have a positive effect on companies' future activities, and stakeholder interests encompass sustainability-related activities [[47], [48], [49], [50], [51]]. Accordingly, the levels of marketing perspective are categorized into three—micro-, meso-, and macro-marketing—to analyze the need for appropriate sustainability strategies and activities for each level, which are also related to stakeholders' interests [19,27,29,42]. Sustainability and marketing are considered significant factors for further investigation [25,42,44,47,48,51]. For example, micro-marketing encourages consumption behaviors through the creation, communication, delivery, and exchange of goods, while meso-marketing focuses on designing sustainable strategies regarding an organization's structure and culture, such as sustainable performance [14,15,42,[52], [53], [54]]. By contrast, macro-marketing embraces the complexity of both micro- and meso-marketing, as both drive sustainable systems and make sustainable contributions to enterprises and industrial companies [42,55,56,58]. Consequently, attempts have been made to develop sustainability views and frameworks from a marketing perspective [7,15,42,55]. However, limited literature is available highlighting the trends in sustainability and marketing based on objective and reliable data.

Sustainability is a natural process in business-related disciplines, especially in marketing, because both company executives and scholars are responsible for a profitable and sustainable future [4,7,31]. Subsequently, there have been numerous definitions of sustainability in the marketing field. Although the scope and definition of sustainability are unclear, studies on marketing generally focus on the environmental sector among the three pillars of sustainability (environment, economy, and society) [17,33,34]. For instance, Chabowski et al. [57] argued that sustainability activities in marketing, as a behavior of green consumers, provide important results based on an understanding of the reality of active green or ethical values regarding concrete purchases. Specifically, they argued that regardless of the broad and diffuse concept of sustainability, consumers are predisposed to purchase sustainable products because their purchasing behavior is constrained by conventional marketing factors such as price, brand, and availability, which enhance sustainable consumption patterns. Hence, the antithesis relationship between marketing and sustainability has been reviewed carefully to identify the significance of one idea compared with the other [6,7,15,19,20,22,30,32]. Sustainable marketing is a strategy that applies general marketing functions, processes, and techniques to services [7,8,32]. This increases the number of sustainable products and services and enhances sustainability-driven consumerism while continuously satisfying customers and other stakeholders [7,19,21,22,[58], [59], [60], [61], [62], [63]]. Sustainable development represents a path to resolving the apparent paradox between profitability, consumption, and the need to protect the environment [64,65]. However, although there are several sustainable applications in marketing, altering the research trends of sustainability and marketing is regarded as less concerning. This is a challenge in the development of new concepts of sustainability and marketing.

The research gaps in the literature on sustainability and marketing can be described in two ways. First, research on sustainability and marketing is limited in the business context [7,19,21,22,[59], [60], [61], [62], [63], [64]]. The literature on sustainability and marketing tends to focus more on micro- and meso-marketing perspectives than on macro-marketing, such as enterprise systems and industrial aspects that would eventually impact marketing activities [36,[67], [68], [69]]. Second, although sustainability and marketing are two significant research themes, meaningful results based on empirical evidence are lacking [4,7,17,70,71]. Owing to the absence of an empirical analysis reviewing and investigating existing literature on sustainability and marketing, the scope of research and strategy development is mainly limited to micro- and meso-marketing perspectives [12,15,19,42]. Moreover, sustainability and marketing are not limited to business-to-consumer relationships, such as the promotion of green marketing to enhance a company's image and profit [34,37,70]. Industrial businesses must consider the perspective of macro-marketing for sustainability. This study addresses these gaps by providing empirical evidence on research trends in sustainability and marketing.

The motivation for this study was to broaden and connect the two mature and significant themes of sustainability and marketing. We employ topic modeling, more specifically, Latent Dirichlet Allocation (LDA), the most widely used algorithm in topic modeling, for effective and systemic analysis of the existing literature on sustainability and marketing. Previous studies have used different methods to investigate the impact of sustainability marketing on consumer behaviors and intentions, such as communication methods (e.g., psycho–physiological and attitudinal measures), surveys, mixed methods (minor qualitative and major quantitative parts), action research experiments (i.e., developing a series of marketing initiatives and testing their impact on consumption behavior), and social invention measurements [1,15,21,26,27,29,35,55,64,72,73]. This study systematizes scientific knowledge and research trends in sustainability and marketing literature over the past 10 years, from 2010 to 2020. This is similar to a recent study by Pizzi et al. [74], which systematized interdisciplinary topics, management, and sustainable development goals (SDGs) to obtain new results. However, the present study is different in that it fills the research gaps in identifying the research topic and its trend over time obtained from a top-ranked sustainability- and marketing-related journal using LDA. To address these research gaps using the LDA method, two key objectives were targeted in this study:

● Investigate and classify existing literature on sustainability and marketing, including macro-marketing aspects, in addition to micro- and meso-marketing perspectives.
● Investigate and discuss the research trends of sustainability and marketing, and the implications of applying the trends for future research and practices.

The aim of this study is to provide scholars, managers, and executives with a clear focus on sustainability and marketing trends over the last decade, while complementing the topics that scholars and practitioners consider in the future. Additionally, this study also discusses topics investigated during certain year(s) in the last decade. Our results demonstrate that particular topics were researched only during certain year(s), with no further significance for continued research in the future.

The remainder of this paper is organized as follows. Section2 describes the methodological approach adopted by the authors. Section 3 consists of the results collected from the LDA topic modeling analysis and an interpretation of the output. Section 4 portrays implications and conclusions of this research.

## 2 Methodology and materials

### 2.1 Research method

**Fig. 1:**
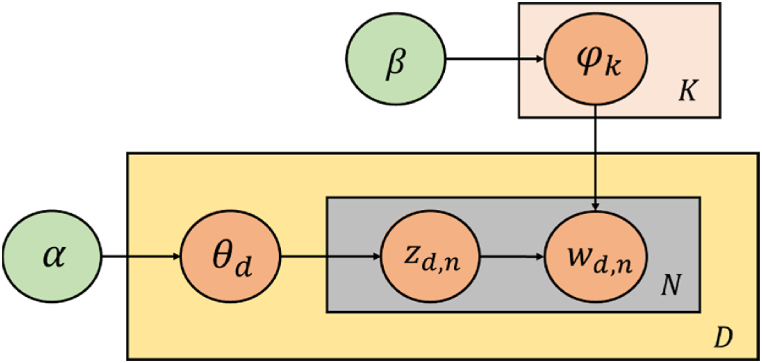
Graphical model of LDA.

Topic modeling is a popular statistic tool for latent variables in large unstructured textual data [75,76]. Each document has a probability that it belongs to a topic, and each topic is identified by a probability distribution of words. In this study, Latent Dirichlet Allocation (LDA), the most widely used algorithm in topic modeling, is used [76]. A document is generated based on the word distribution of a topic and how topics are mixed in the document. Fig. 1 shows the document generation process of LDA.

Each node of Fig. 1 represents a random variable, and α and β are hyperparameters of the Dirichlet distribution φ_k_ and θ_d_. φ_k_ is distribution of words belonging to the kth topic, and θ_d_ corresponds to distribution of words belonging to the dth document. The topic z to which each word belongs is determined by θ_d_. w is a word generated based on θ_d_ and z_(d,n)_. This generation process is iteratively performed for N words included in M documents. For the LDA algorithm, α, β, and K must be determined by the user. φ_k_ and θ_d_ are learned by LDA.

**Fig. 2:**
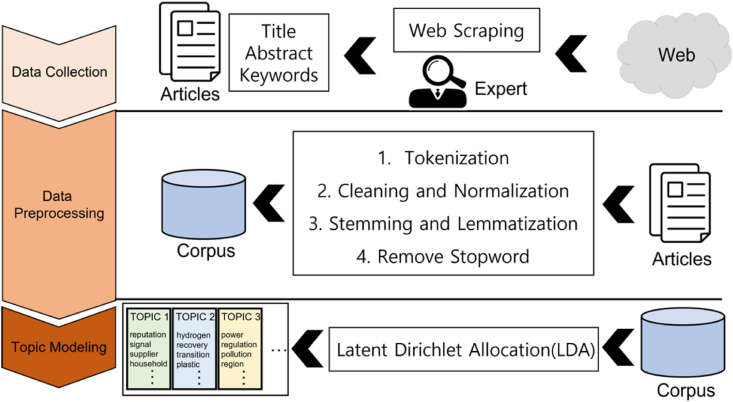
Topic model building process.

Topic modeling is a proven analytical method of trend analysis in several research fields such as big data in marketing [77], transportation [78], personal information privacy [79], competency-based education [80], emergency medicine [81], social media and sustainability [23], and physics education [82]. The analysis method is standardized with literature collection, pre-processing, and topic modeling. Fig. 2 shows the data collection and pre-processing for topic modeling in our model. Sections 2.2 and 2.3 describe the data collection and preprocessing process in detail.

### 2.2 Data

We collected sample data for systematizing the scientific knowledge and research trends of sustainability in the marketing field. Based on the Scimago journal rank indicator that measures scientific impact in the marketing, strategy, and management categories, ten journals among the top 100 journals related to sustainability indexed by SCIE or SSCI were selected. Since the subject of all papers in the selected journal is not sustainability, the title, abstract, and keywords were designated as ‘sustainability’ or ‘sustainability’. Additionally, since ‘market’ and ‘marketing’ are words that appear frequently in articles in the marketing field, they must appear in the title, abstract, or keywords. Although ‘market’ or ‘marketing’ does not appear directly, it does include the appearance of the words ‘customer’, ‘consumption’, ‘retail’ and ‘retail'.

**Fig. 3:**
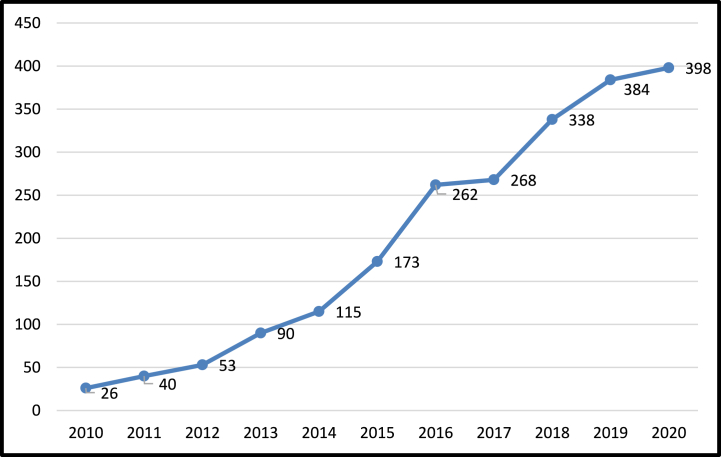
Number of sustainability and marketing articles.

In 2012, the United Nations Conference on Sustainable Development agreed on the process of selecting the Sustainable Development Goals to be implemented from 2015 to 2030 [83]. Therefore, it is expected that interest in sustainability in the marketing field will increase continuously, and based on this, data has been collected since 2010. Moreover, Fig. 3 depicts the change in the number of articles collected yearly. The number of sustainability articles in marketing steadily increased from 2010 to 2020, indicating a progressive interest in the field. Data for 2021 were excluded as it may not be suitable for trend analysis due to COVID-19.

Table 1 shows the number of sustainability and marketing articles selected in each journal containing the selected keywords. The journals in Table 1 are listed by the number of samples, and the numbers in Table 1 do not indicate the ranking. The Journal of Cleaner Production publishes more papers each year than any other journal, and we have been able to collect more data from this journal.

**Table 1:** The number of sustainability and marketing articles from 10 journals.

No.	Journal name	Approximate number of Published Articles	Number of Selected Articles
1	Journal of Cleaner Production	30,000	1957
2	Journal of Business Research	4300	87
3	Journal of Marketing Management	800	38
4	Psychology and Marketing	900	19
5	Journal of Marketing	500	14
6	Fashion and Textiles	200	9
7	Journal of Supply Chain Management	250	8
8	Journal of Retailing	400	8
9	Journal of Marketing Research	750	4
10	Journal of Interactive Marketing	250	3

To obtain meaningful results in LDA analysis, a sufficiently large size of text corpus is required. However, there is no exact guideline on the size of the corpus, and Schmiedel et al. [84] suggested that it is difficult to analyze the corpus with less than 100 documents. In addition, they suggested that analysis is difficult even when the number of documents is small, including long documents. In this study, only the title, abstract, and keywords of 2147 papers are used for LDA analysis.

### 2.3 Preprocessing

Before the LDA analysis, the text corpus is preprocessed. First, all text in the corpus is converted to lowercase. After removing the special characters, the text is tokenized by applying word tokenization.

By verifying the part–of–speech of each token, only nouns are extracted, and the number of tokens is reduced by extracting headwords. Finally, words such as study, paper, and research, that frequently and meaninglessly appear in the abstract of academic documents are defined as stop-words, and the corresponding tokens are removed.

For LDA analysis of the data obtained from the preprocessing, we determined the number of topics, alpha, and beta, which are hyperparameters of the Dirichlet distribution. After LDA analysis was performed by varying the number of topics to 5, 10, 15, and 20, the number of topics was 15, determined by examining the word distribution of each topic; alpha and beta were set to 0.1 and 0.01, respectively.

## 3 Results of topic modeling and discussion

### 3.1 Topic identification

The result of topic modeling highlighted the novelty of this study by analyzing the title, abstract, and keywords of 2147 articles from the journals that are indexed in either SSCI or SCIE. Moreover, the identified topics are not limited to micro- and meso-marketing, but expanded to macro-marketing perspectives including the importance of industrial aspects in sustainability and marketing disciplines. For instance, rather than focusing on business-to-consumer relationship and business-to-business keywords, we obtained keywords ranging from material processing to consumption, and discussed in detail in the following sections.

This study obtained 14 topics by running LDA based on two types of posterior probability distributions, which are the topic distribution of each article and the relevant word distribution of each topic. Table 2 shows the list of these 14 topics including the top 10 words shown for each topic and the shares of the topics in the entire corpus. Topics were arranged and numbered according to the order that the LDA derived. The LDA indicates topics by algorithm; however, it does not provide a specific name for each topic because authors are required to label the topics. The authors of this article labeled each topic through discussion and literature analysis of the entire content of the top 10 words and the highest loaded words for each topic.

**Table 2:** Obtained topics from the LDA.

Topic	Top 10 relevant words	Share
(T1) Sustainability performance	reputation, signal, supplier, household, climate, power, dimension, report, category, lubrication	0.0317
(T2) Product sustainability	hydrogen, recovery, transition, plastic, researcher, generation, health, topic, asphalt, fermentation	0.0359
(T3) Environmental sustainability	power, regulation, pollution, region, heat, network, air, loss, temperature, urbanization	0.1160
(T4) Sustainable development	behavior, concrete, strength, choice, property, intention, gap, emotion, utility, wood	0.0503
(T5) Sustainable urban entrepreneurship	construction, input, entrepreneurship, aggregate, steel, land, variable, stage, home, measure	0.0429
(T6) Sustainability in luxury fashion consumption	luxury, behavior, customer, intention, CSR, fashion, attitude, purchase, apparel, orientation	0.0512
(T7) Sustainability of industrial projects	oil, degrowth, project, biofuels, ethanol, security, state, capability, transportation, construction	0.0340
(T8) Sustainability scenario of industry	machine, scenario, electricity, power, phase, part, metal, plant, generation, element	0.1383
(T9) Sustainability drivers and barriers	adoption, barrier, treatment, wastewater, part, productivity, advantage, attribute, profitability, leather	0.0484
(T10) Enterprise sustainability	criterion, enterprise, parameter, investment, optimization, rate, location, subsistence, selection, field	0.0708
(T11) Eco–design paradox	group, crop, risk, matrix, satisfaction, eco–design, action, park, education, institution	0.0582
(T12) Transportation sustainability	city, vehicle, greenhouse, distance, logistics, transport, choice, price, wine region	0.1658
(T13) Innovative sustainable network	innovation, network, government, customer, risk, driver, concept, advantage, goal, example	0.1001
(T14) Sustainability in fashion community	brand, fashion, community, purchase, gap, perception, organization, shopper, disposal, medium	0.0564

### 3.2 Topic analysis

14 classified topics elevated the view of the research in sustainability and marketing field. Topics were grouped based on the three pillars of sustainability (that is, social, environmental, and economic goals), which were closely enlaced with one another but not mutually exclusive. T13 and T14 were categorized as social pillar, and T1, T2, T3, T4, T9, T11, and T12 as environmental pillar. Because the rest of the topics were categorized as economic pillar, T5, T7, and T8 included the expansions of existing industrial business fields. Also, T6 included fashion marketing while T10 included management. We classified 14 topics on sustainability and marketing, and highlighted grounds distinguished from general sustainability and marketing research. Each topic was named based on the proportion of 10 keywords that are statistically significant and related literatures regarding both sustainability and marketing.

This study compared its findings to that of Al amosh and Khatib [49], Anwar and El–Bassiouny [85], Bhattacharyya [7], Effah et al. [86], Jones et al. [30], Lunde [17], Pizzi et al. [74], and Su et al. [29] which reviewed and reported the research trends of sustainability and marketing based on the relationship between sustainability and marketing. They debated the impact of sustainability and marketing on each other, and identified four factors related to sustainability regarding the goal of marketing: demography, technology, values, and government. The demography factor refers to human potential (example, migration phenomena); technology factor refers to the development of technology through company's system; value factor refers to a system of values shared in social and market activity by stakeholders, companies, employees, authorities, consumers, etc.; and government factor refers to the policies of federal and local authorities toward society and economy. The identified factors correspond to the 14 topics that the study outlined (particularly T5, T6, T12, and T13). Analysis of each topic is provided below, focusing on the relationship between sustainability and marketing, holistic perspective of business including marketing.

(T1) Sustainability performance

Sustainable performance assessment has attracted scholars in the last decade, because it fosters the connection between environmental, social, and economic performances of companies [[87], [88], [89], [90]]. Sustainable performance is an approach that balances human to non–human associations through more responsible management models and sustainable marketing [57,91,92]. Scholars emphasized that sustainable and business performance have a significant relationship after reviewing each component of sustainability [12,89,91]. For environmental performance, companies must identify the sources of environmental problems regarding production, procurement, and transportation [12,16], and the ability to alleviate pollution, waste, environmental accidents, and the use of hazardous materials would indicate the business performance [[93], [94]]. For economic performance, reducing cost of purchased materials, waste treatment, waste discharges, energy consumption, fines for environmental issues, and profitability and sales [95] would indicate the business performance related to sustainable performance. However, when sustainable performance becomes the aim of business, research indicates that companies may face high costs of environmental–related practices which result in profitable performance [96,97]. For social performance, companies are required to be socially responsible and stakeholder communities are required to approve the activities that companies perform to maintain their relationship [12]. Hence, increasing awareness of companies' social performance will develop its positive image and strengthen relationship with stakeholders [98]. Marketing involves fulfillment of companies’ needs with minimal negative impact on sustainable performance by supporting sustainable promotion, price, and distribution process [99,100].

(T2) Product sustainability

From the top 10 words of product sustainability, “recovery” is the most related word for this topic. Accordingly, research and product designers asked questions such as “how can products be designed and produced to be inherently good for the future of the planet and users?” [101]. In a business perspective, keys of product sustainability are three basic Rs (reduce, reuse, and recycle), and other three Rs (recover, redesign and remanufacture) [102,103]. It is not only about production but provision of sustainable benefits and maintenance of the environment after full life cycle of products from raw materials’ extraction, use, disposal and eventual reuse [103].

Generally, product sustainability considers how products can provide sustainably balancing contributions of products, thereby creating multiple shared values for stakeholders [104]. From a business perspective, product sustainability keeps companies competitive by providing differentiated products. This increases consumers’ satisfaction, which indicates economic success [104]. Therefore, marketing strategies which suggest the current level of product sustainability, market understanding, holistic improvement of the market, good products over better products, and societal value over product value from the idea of product design, should be developed [101,104].

(T3) Environmental sustainability

Environmental sustainability gained its importance in the last decade [105]; however, it has not been achieved owing to overconsumption of materials, emission of hazardous pollutants, and indiscriminate use of energy [106,107]. COVID–19 pandemic indirectly has a negative impact on maintaining momentum for environmental sustainability because it struck down numerous companies. Therefore, they are less likely to perform any environmental sustainability activities at both local and global levels [108]. Because the environment is not something that any type of company can control, companies are required to adopt green business practice (GBP) by engaging in environmentally friendly actions which may be exposed later by governments and stakeholders to reduce less eco–friendly sources from companies' activities [[108], [109], [110], [111]]. Moreover, marketing enhances companies' values based on ecological modernization theory by providing appropriate marketing roadmap of environmental sustainability: 1) increasing renewable resource consumption, 2) decision making regarding long–term consequences, 3) avoiding damages on ecosystem, 4) focusing on people's welfare, and 5) avoiding excess pollution [105]. Stakeholders' engagement in the steps on the roadmap is the key to enhancing the marketing impact of environmental sustainability of companies [110].

(T4) Sustainable development

Sustainable development refers to the search for harmony among the environment, social, and economic activities [91,112]. In this study, some relative words for this topic are emotion and behavior based on the proposition. This is in line with studies which argue that sustainable development is related to humanity and overconsumption behaviors [113,114]. Studies have revealed that emotions play an important role on people who engage in sustainable behaviors [115], with the exception of recognition [114]. Regarding sustainable development, emotions are perceived with two kinds of behaviors such as self– and others’–behaviors. Pride and guilt are self–behaviors, while respect and anger are others' behaviors [116,117]. However, emotions related to sustainable development are not limited to these four kinds of emotions [116] because people tend to experience them when they are aware that they realized or failed to live up to an ideal or actual self–representation [118]. Sustainable development related emotions are more complex than basic emotions because those emotions are considered as self–conscious emotions that are elicited after flouting social norms or personal based standards [119]. Additionally, changing human behavior is raised as a strategy of priority for sustainable development [120,121]. Luetz et al. [121] argued that an important feature of changing human behavior for sustainable development would be the desire to become pacesetters in the world and leave a positive legacy. Therefore, to support and sustain human behavior change, development of positive emotions to cultivate happiness must be done first [122,123].

How can marketing impact sustainable development regarding emotions and behaviors of people? The role of marketing is to understand and change consumers' behavior by influencing their attitudes and beliefs [30]. Proper marketing strategies focusing on stimulating people's emotions regarding sustainable development should be suggested to increase people's sustainable behaviors including thorough understanding of their consumption processes [30,91]. Hence, effective and efficient marketing strategies which attains a balanced approach to socio–economic development based on a strong respect and understanding for ecological systems [30,124,125] are suggested.

(T5) Sustainable urban entrepreneurship

Sustainable urban entrepreneurship is considered opportunities to incorporate and develop efficient and innovative ideas connected to sustainability, thereby emerging in urban society system and forming urban networks [126,127]. Sustainable entrepreneurship depends on individuals’ capacities to transform ideas into actions such as create and innovate a process of identifying latent opportunities that create economic, environmental, and social values [128]. Hence, sustainable entrepreneurs are required to make impact on urban entrepreneurship, local economy, and social and environmental system by stimulating the formation of businesses and networks of towns and cities through acquiring tangible and intangible resources and impressing investors [127]. Korhonen et al. [129] highlighted that in favoring sustainable urban entrepreneurship, the circular economy for global sustainability is important because urban entrepreneurship is part of creative economy. Hence, sustainable urban entrepreneurs in towns and cities would utilize marketing to support themselves through creative and cultural activities which may generate new businesses and jobs, and create new partnership and networks, both profit and non–profit. The three goals of sustainable urban entrepreneurship are participation in circular economy, local economic growth, and well–being of the towns and cities [124], and sustainable urban entrepreneurs are required to understand.

(T6) Sustainability in luxury fashion consumption

In fashion industry, sustainability is a mandatory topic for researchers and practices [[130], [131], [132]]. Sustainable fashion consumption behavior is defined as a variety of behaviors that consumers are willing to minimize regarding their fashion consumption decisions [130]. It has been alarming especially in fast fashion industry because of the life cycle of fast fashion products, including products acquisition and disposal, are short with no sustainable plans to reduce any negative impacts [132]. Harris et al. [133] argued that consumers should be exposed to more sustainable and higher quality fashion products to achieve sustainable fashion consumption, in which fast fashion business pursues more trendy styles with affordable prices and low–quality materials that end up to short life–cycle of products [134]. In luxury fashion consumption, different perspectives on consumers' consumption behavior should be addressed because consumers look for self–congruency, timelessness [135], not trend–led but durability from luxury fashion brands [136], which indicates that luxury fashion consumption is a high–involvement consumption [137]. Moreover, luxury fashion consumption is heavily influenced by context and social conventions [138,139] because most of luxury fashion products are becoming less wasteful and exclusive; however, they contribute to humanity's expression of their own values [140]. Therefore, in luxury fashion consumption, sustainability is one of the main marketing strategies because sustainable brand image would differentiate one brand from the other to increase competitive advantage in the fashion market [131]. Additionally, educating consumers about sustainable motivations and consumption may be done through marketing strategies of luxury fashion brands which develops a personal connection with consumers; this influences consumers' purchase intentions positively [130,136].

(T7) Sustainability of industrial projects

Sustainability of industrial projects requires the following characteristics: 1) acceptable consumption level of energy, reduced waste product rate, restriction of urban expansion (environmental pillar); 2) low–cost demands and their influence on markets (economic pillar); and 3) impact on cultural and natural herniate including employment (social pillar) [141]. To guide decision–making in sustainable industrial projects, knowing what the expected impact of industrial projects is on the relevant sustainability concerns is essential [142]. The impact which industrial projects managers should consider are three folds: 1) changes that people can perceive immediately within current projects; 2) impacts that are from the use over time including the opportunity to consume less/more resources, and 3) persistent changes perceivable at macro–level such as structural economic and people's behavioral changes [142,143]. To pursue sustainability of industrial projects, economic performance, compliance with regulations, and utility of resources and materials are considered throughout the entire industrial projects [16,141,143]. Furthermore, in sustainability, industrial projects should cover all three dimensions in the context of industrial growth.

(T8) Sustainable scenario of industry

Recent studies about sustainable scenario of industry mentioned a common concept, Industry 4.0 [[144], [145], [146], [147]]. Scholars expected that information technology and social media will show astounding growth that will influence people's perception on products and industries [144,148]. Key features of Industry 4.0 are interoperability, information transparency, technical assistance, and decentralized decision making [145]. For the interoperability feature, machines including devices and sensors, and humans are connected to and can communicate with one another, while the information transparency feature is a system that creates a virtual copy of offline world through data to contextualize information. The technical assistance feature shows the ability of the human supporting systems to assist humans with tasks that are too dangerous and/or difficult, and the decentralized decision–making feature the ability of physical and cyber systems to make decision easily on their own and become as autonomous as possible. These features are based on technologies of the industry that lead to collaborative communication systems either with machines or people and reduce labor turnover; this addresses social pillar of sustainability [147,[149], [150], [151]]. Social perspective in the scenario of Industry 4.0 creates safe work environment for employees to reduce accidents in the workplace and boost their morale [152,153]. Economic and environmental aspects of sustainability in the scenario of Industry 4.0 are integrated and interconnected value networks as cost reduction through efficient utilization of resources, and better performance in the market are connected to each other [147,151]. Specifically, environmental scenario of Industry 4.0 concerns efficient utilization of energy and reduction of CO_2_ emanation when energy saving is directly proportional to productivity [149], and when economic scenario considers improving stock and distribution center administration [154]. Hence, scholars and practices are aware that sustainable scenario of Industry 4.0 should be implemented in the industries. However, implementing process would vary based on companies' and industries' project scenarios regarding different organization structures or sizes, which makes it challenging especially to those who are experiencing shortage of economic resources [146]. Considering sustainable effects and benefits of Industry 4.0, industry experts are required to consider implementing Industry 4.0 comprehensively in industry scenarios [146].

(T9) Sustainability drivers and barriers

There are drivers and barriers of sustainability that obviously have different impact on sustainable construction. Drivers of sustainability are defined as a response to balance the pillars of sustainability [155,156]. Drivers vary based on the country or region; this is because they have different priorities and beliefs in the implementation of sustainable practices [157,158]. For example, in Chile, drivers of sustainability are cost reduction, company awareness, market differentiation, and regulations [159], whereas the USA considers resource and energy conservation, waste reduction and improvement in indoor environmental quality as the most significant sustainability drivers [160]. Drivers are grouped into three factors similar to the three pillars of sustainability, and the most influential factor is the environmental factor, followed by social and economic factors [156]. Barriers makes industries need a considerable amount of time and effort to adopt sustainability; in developing countries, barriers make sustainability impossible or unprofitable to adopt [161]. Barriers are grouped into factors such as cost, knowledge and information, workforce, government, client and market [156,162]. Cost and government related issues contribute a significant proportion of sustainability barriers such as lack of government promotion and incentives [162]. Of course, it is not feasible to pay equal attention to both drivers and barriers simultaneously; however, developing suitable strategies focusing on critical drivers and barriers will enhance sustainability issues in the market and industries [163]. In the case of barriers, marketing does not play a substantial driver role because its influence and the level of market orientation act as conditions for sustainability [158]. For barriers, however, creating awareness of sustainability among the market and the industries would show significant results such as reduction of negative attitudes and increment in supportive attitudes toward the companies [163].

(T10) Enterprise sustainability.

Enterprise sustainability is about balancing the three pillars of sustainability in the performances of the enterprise as today's companies are looking forward to creating sustainable values, such as harmonizing profitability and natural resources with energy consumption [164]. However, current enterprise systems are complex, and this makes it more difficult for companies to address sustainability issues. Additionally, uncertainty of economic situations has an impact on investment and profitability [164,165]. As investment behavior directly influences economic growth and government policies, enterprises are required to make comprehensive assessment and actively adapt investment and management processes under the condition of uncertainty to maximize profit and reduce losses [165]. Moreover, enterprise sustainability should be evaluated according to the appropriate platforms such as different spatial (local, national, and international.) and temporal (short– and long–term) levels [166]. Hence, there are extrinsic and intrinsic reasons that enterprises opt for sustainability. The extrinsic reasons impact positively on the world by minimizing adverse effects on the environment while the intrinsic reasons increase turnover through higher stock prices and production innovation, and reduced costs through energy saving [166,167]. Therefore, enterprise sustainability is formed with focal firm, supply chain and context which produce win–win outcomes even though pursuing enterprise sustainability may require difficult trade–offs [167].

(T11) Eco–design paradox.

Eco–design is the dominant model of product sustainability [104] and is defined as the integration of environmental aspects into product design and development, to reduce adverse environmental impacts throughout a product's life cycle (ISO 14006). Scholars and practices agree on the idea of sustainable products to optimize products' environmental sustainability using eco–design [104]. The goals of eco–deign help to meet consumer's requirements by focusing on improving products' environmental performances and minimizing its negative impact on the environment [168]. Specifically, scholars argued the goals of eco–design are durability, material efficiency, the restriction of problematic materials, energy efficiency, and efficiency in use such as recyclability and reparability [168,169].

Design paradox addresses limitations in the specific stages of process development and some groups of people involved in the stages, owing to lack of knowledge [170,171]. Eco–design paradox is defined as a divergence between possible environmental improvement of development period and product knowledge [170,172,173]. It is mostly about the environmental pillar because eco–design features are environmental assessment of products, environmental improvement, and both features at the simultaneously [174], which requires stakeholder engagement across entire product life cycle and spans [175]. Although designers are aware of the need of a holistic approach for eco–design, integrating eco–design into existing systems is challenging because most companies lack the systems to achieve eco–design goals and maintain the continuous improvement in the development process, in which eco–design paradox is more realistic than the concept of eco–design for the companies [169,170,176]. Scholars asserted that achieving holistic approach of eco–design requires a large amount of knowledge both in product development and environmental improvement for companies especially within the design and supporting teams such as management and marketing [177]. Nevertheless, eco–design paradox is perceived as an overlooked research topic [103].

(T12) Transportation sustainability.

Transportation sustainability is the importance of access, efficiency, affordability and lowered negative environmental impacts [178]. Strategies of transportation sustainability supported by the government and public are shifting from the use of private cars to public transportation services, walking, and cycling [179]. However, research about transportation sustainability argued that universally accepted definition of transportation sustainability should be given to the public transportation authority personnel prior to execute appropriate transportation sustainability strategies [178,180]. Hence, a globally accepted definition of transportation sustainability is “the one that meets better accessibility to individuals and societies with consideration of the ecosystem and efficiency of a combination of transportation modes” [181].

The environmental pillar of transportation sustainability is mainly focused on eliminating negative outputs of transportations such as decreasing greenhouse gas emission, air pollution, waste and noise pollution followed by efficient use of resources and materials to improve its environmental performances [178,181,182]. The social pillar of transportation sustainability is the most complex and challenging dimension to identify because it deals with overlapped elements with the economic pillar such as safety, socio–economic and physical access, information availability, attractiveness, coordinated management, commitment to plans (example, public participation), health, and aspects of governance (example, regional cooperation) [178,181,183]. Essential elements of the economic pillar of transportation sustainability are ridership, costs to service provider, volume of transportation, financial stability, fare revenue, and operational efficiency [178,183]. For public transportation sustainability, developing strategies must consider two agents, urban form and governance, as they create the conditions for the public transportation [184]. Specifically, urban forms consider the physical form and land use, land area and density, and centralities and regionalism while governance deals with regional integration, funding and finances, and long–term goals of public transportation [178,181,182]. Besides public transportation, forms of transportations such as freight and rail have agents incorporating with transportation sustainability; however, they share common implications that are a better modal balancing, future economic development, quality transport, and environment and social well–being of the entire society [178,181,[183], [184], [185]].

(T13) Innovative sustainable network

Innovative network requires multiple participants who are willing to be part of a dynamic and complex unlimited system and is formed by industry research university cooperation and industrial association and enterprise alliance [186]. Innovative sustainable network is the environmental, social, and customer values in the concepts of the network [186,187]. The most familiar form of the innovative sustainable network is the circular economy, which promotes sustainable development by improving economic, social and environmental impacts [188,189]. Braz and de Mello [189] focused on the concept of the innovative sustainable network on supply chain network, in which the goal of innovative sustainable supply network is to contribute to the achievement of the sustainable development goal of production and consumption [190]. Chen and Chiu [187] interpreted the innovative sustainable network with the value chain perspective focusing on service–dominant system, which is also called product–service system (PSS), that integrates products, services and networks as a whole to create a positive impact. According to the aspects of innovative network, PSS would be the most suitable form of innovative sustainable network because its platform also requires open systems and is formed with enterprise alliance, industrial association, and the industry research university cooperation, for proper operation [186,187]. Moreover, PSS supports continuous competitiveness among companies, customer satisfaction and alleviates negative environmental impact because sustainability is the main value of PSS [191]. Because the sustainable value should equally be considered in the innovative sustainability network, three pillars are needed to be incorporated in the innovative network as well.

(T14) Sustainability in fashion community.

A general concept of community is a group of people who voluntarily participate in forming social connections with community members for a certain object on online and/or offline platform [192,193]. In fashion community, people share information and knowledge about entire sectors of fashion such as brands, products, and promotion events [[192], [193], [194]], which shows strong connection between communities and product proliferation [195]. Sustainability in fashion community means that people in the fashion communities are actively interacting and creating contents regarding sustainable fashion products, branding, and consumption, including ethical fashion management, and CSR of fashion brands and companies [60,193,194]. The fashion community with strong sustainability focus emphasized socially shared meaning of pro–environmental behaviors and ethical fashion consumption behaviors [193,195]. On the social media community, sustainable fashion is promoted because numerous individuals including celebrities who support eco–friendly, ethical, and fair–traded fashion, use their cultural capital, for example, to question anti–fur fashion products [193,196]. Those who are in the fashion community but show less or no support to sustainability in fashion, argued that eco–friendly fashion is paradoxical in nature because fashion is one of the most unsustainable industries with inefficient product life–cycle when eco–friendly fashion promotes durability and recycling of products to be sustainable [197]. Scholars investigated fashion communities regarding this issue and reported that members of the communities are aware of sustainability; however, their focus is changing from environmental issues (example, energy waste, pollution) to eco–friendly fashion products (example, eco–friendly fabric and design, and consumption) [193,197] by sharing knowledge and perspectives regarding entire fashion product life cycle.

### 3.3 Topic trends analysis

#### 3.3.1 Topic trends over time

**Fig. 4:**
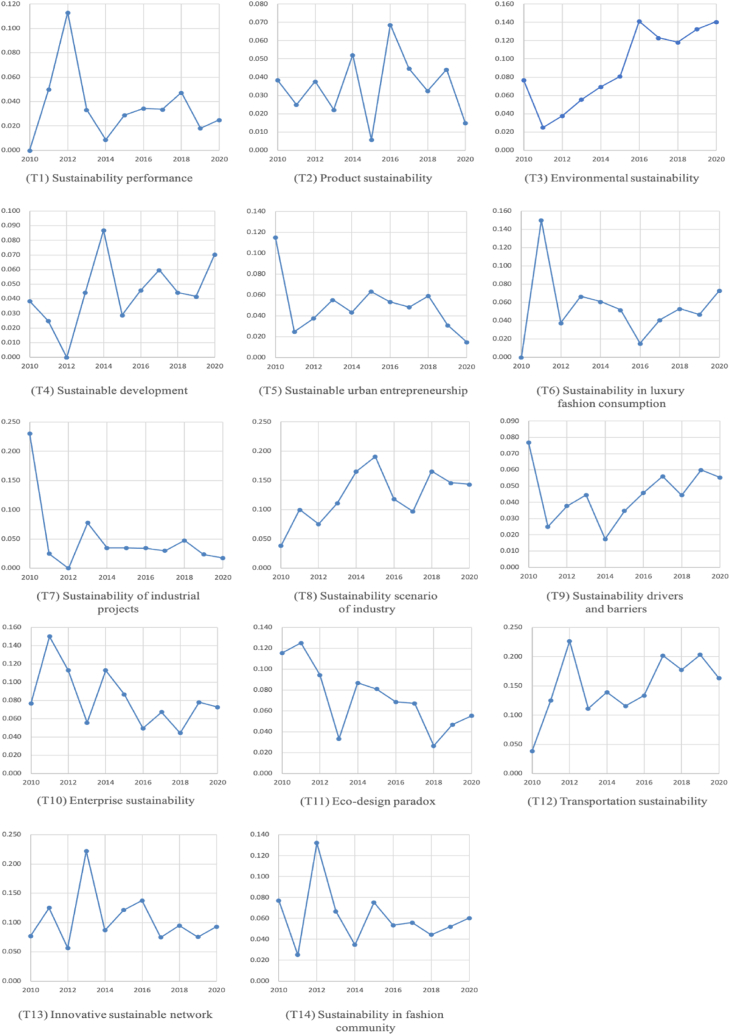
Research topic trends of 2010–2020 (OLS).

Based on the LDA results, this study distinguished between the topics that were actively researched (that is, hot topic) and topics that did not attract interest (that is, cold topic) over time [32,198,199]. Finding the trends in research topics is one of the most attractive efforts of this study. We made this difference by observing the changes of each topic's weight over time. Fig. 4 shows the weights of changes of the 14 topics over time.

A linear regression model was employed for each topic with the topic weights in corresponding years as dependent variable, and time as an independent variable. In order to minimize cost function for defining α and β in the linear regression equation, we adopted Ordinary Least Square method (Eq. (1)). Estimated linear regression equation (Eq. 2) where θ_*jt*_ is average share of topic *j* in time (year) *t*. The key component of interest in this research is the coefficient $\beta_{j}$. If this value is significantly positive (negative), the topic was indicated as a hot (cold) topic (Table 3). Consequently, two hot topics and one cold topic were identified when *p* value is less than 0.050: T3 (hot topic), T8 (hot topic), and T11 (cold topic).

(1) $$\min\sum_{j=1}^{n}{\left(\theta_{jt}-\alpha_{j}-\beta_{j}t\right)}^{2}$$

(2) $$\theta_{jt}=\alpha_{j+}\beta_{jt+}\varepsilon_{j}$$

**Table 3:** Topic types and regression results.

Topic	Slope (a1000)	*p–*value	Type
(T1) Sustainability performance	−1.5719	0.6058	•
(T2) Product sustainability	0.0565	0.9754	•
(T3) Environmental sustainability	10.8981***	0.0007	**Hot**
(T4) Sustainable development	3.1692	0.1603	•
(T5) Sustainable urban entrepreneurship	−3.7853	0.1396	•
(T6) Sustainability in luxury fashion consumption	−0.8952	0.8209	•
(T7) Sustainability of industrial projects	−9.3312	0.1236	•
(T8) Sustainability scenario of industry	8.2058^a^	0.0462	**Hot**
(T9) Sustainability drivers and barriers	0.934	0.5850	•
(T10) Enterprise sustainability	−5.0408	0.0962	•
(T11) Eco–design paradox	−6.9706**	0.0105	**Cold**
(T12) Transportation sustainability	8.7754	0.0820	•
(T13) Innovative sustainable network	−2.2567	0.6309	•
(T14) Sustainability in fashion community	−2.1873	0.4452	•

a *p* < .05, **p < .01, ***p < .001. Slope is multiplied by 1000.

From the results, this study reveals that the research trends across the sustainability and marketing fields have become more diverse and detailed. Specifically, this study revealed detailed applications in different sectors of various business types rather than providing general topics regarding sustainability and marketing. For instance, T2 and T4 show statistically insignificant weight change, which corresponds to the general discussion topic of sustainability research that deals with harmony between social, economic, and environmental activities, such as reducing the ecological footprint from manufacturing process. However, T3 shows significantly positive weight change and has been represented as a hot topic, which corresponds to specific component of sustainability, environment, and continuous interest of scholars toward the environmental ecosystem. On the contrary, T11 shows significantly negative weight change, which corresponds to specific application of sustainability in product design and life–cycle assessment of product, and have emerged as cold topics.

Among the five topics closely related in the economy pillar of sustainability T5, T6, T7, T8 and T10, which correspond to the general discussion of sustainable business sector did not show significant proportion weight change over time. Conversely, T8 emerged as a hot topic, which could be interpreted as a reflection of interest growth of industries’ sustainable scenario, Industry 4.0, which considers collaborative systems between humans and machines to create a solid foundation of sustainable long–term success of industries.

The flow of research shows that the topic trend shifted to more specific topics of sustainability, such as sustainability to environment, product to product design process. Since 2010, research trends have shifted attention from general ideas of sustainability and sustainable development to more environment focused and collaboration of market actors including innovative strategies, such as interaction between humans and machines. Meanwhile, companies faced consumers who are familiar with the idea of sustainable consumption and willing to consume products (example, luxury fashion products) than in the past. Consumers are becoming smarter than before as much as tracking companies' sustainable footsteps, which reminds companies to develop critical marketing strategies regarding their consumers’ sustainable lifestyles.

This study has dealt with sustainability and marketing rather than sustainability in general as continuous debate of the impact of sustainability and marketing on one another. Hence, we expected significant results from either T6, T10, or T14 regarding both sustainability and marketing perspectives. Unfortunately, research trends between 2010 and 2020 showed insignificant weight changes of topics T6, T10, and T14. Therefore, creating and increasing companies’ values (T6), pursuing and promoting sustainable consumption (T10), and connecting sustainable images to fashion brands (T14) would be more than trends because these topics have been continuously investigated and are still on debates among scholars.

A topic's incline and decline could reflect the impact of marketing. For example, promoting Industry 4.0 features for the sustainable scenario of industries may require proper marketing strategies while emphasizing included sustainable concepts. Conversely, the role of marketing in sustainability scenario of industry may be indispensable. Also, environmental sustainability requires a strong role of marketing for reducing pollution through green business practices and stakeholders' engagement at both local and global levels.

In the case of T11, it has become a cold topic, that is, eco–design paradox lost significant popularity, and there is little or no chance that marketing would play a vital role in its success. Although both research and practice agree on the need for considering sustainable product life cycle, awareness of eco–design in the design industry is low because implementing sustainable product design in organizations is practically limited. Therefore, divergence between possible environmental improvement of product development time and lack of product knowledge (that is, eco–design paradox) could reduce the sustainable interest regarding the topic. Hence, this decline reflects the fact that the weight of “eco–design paradox” researches, that also deal with marketing issues, has fallen, rather than a decline of “eco–design paradox” research itself.

#### 3.3.2 Topic proportion over time (year 2010–2020)

In this section, we identify topic proportion for certain years irrespective of hot and cold topics. We simply identified topics that show the highest proportion for certain years, and we intended to provide a hint for future research directions regarding why certain topics were considered only for certain years [1,26,64].

Appendix shows the proportion of the topic for each year from 2010 to 2020. The top topic with highest portion for each year is highlighted in bold and certain topics have been researched continuously for two years or more. Specifically, the analysis reveals sustainability and marketing research aims and scopes of each year focusing on some topics more than others since 2010.

During the early period of 2010–2020, sustainability and marketing research paid attention to different topics each year. In 2010, T7 received the most attention (23.1%), T6 (15%), and (T10) (15%), show equally high proportion among the 14 topics in 2011, suggesting that sustainability and marketing scholars focused on these two topics on this specific year. Moreover, T12 (22.6%) and T13 (22.2%) received the most attention in 2012 and 2013, suggesting that with increasing attention to sustainability, scholars started to pay attention to the combined idea of sustainability and transportation which provides efficient and sustainable transportation services to consumers. Since 2013, ideas of innovative networking and sustainability were considered because industries were promoting their transformation to digitalization and intelligence and networking. In 2014 and 2015, T8 showed the highest portion among the topics, 16.5% and 19.1%, in which the scholars began to be more innovative and the industries were progressing from traditional system to innovative and sustainable systems such as Industry 4.0 features. Although T3 (14.1%) was the most researched topic in 2016, T12 became the top topic from 2017 to 2020. The results from 2017 to 2020 show that both research and practice are ready to move to the next level of transportation including types of transportation such as private car and public bus, to be more sustainable.

## 4 Conclusion, implications, limitations and future research

Our current LDA topic-modeling study for the years 2010–2020 has a two-fold agenda. First, we aimed to investigate and classify the existing literature on sustainability and marketing, including macro-marketing aspects, in addition to micro- and meso-marketing perspectives. Second, we investigated the ideas that scholars and practitioners should consider in sustainability and marketing, based on topic trends over time. We show that understanding and adopting sustainability and marketing trends are critical according to stakeholder theory [25,44,[47], [48], [49]]. For instance, to satisfy stakeholders’ understanding and acceptance of what topics they are interested in, or to have the most impact on stakeholders. Hence, to provide empirical evidence, we developed a topographic map of sustainability and marketing research based on 14 latent topics by utilizing text-mining technology and LDA, which provided originality and differentiation from general sustainability and marketing research.

We identified research topic trends by reporting hot and cold topics based on the measurement of variations in topic distributions over time. From this study, the center of sustainability and marketing research has shifted from general sustainable behaviors or concepts to more environmental and innovative technology. This is illustrated by the rise of T3 (environmental sustainability) and T8 (sustainability scenario of industry), which were identified as hot topics, while T11 (eco-design paradox) was identified as a cold topic. The rise and fall of these topics may reflect the relative strength of the marketing impact on each area. Certain topics exhibited characteristic trends in certain years; in particular, T12 had continuous research interest in the last four years. The result of topic proportion yearly (2010–2020) is irrespective of the trends of topics (hot or cold) because it simply shows the proportion of topics yearly, rather than performing static analysis. However, it shows the importance of certain topics in certain years, demonstrating the unique aims and scope of sustainability and marketing studies for a particular time, and possibly playing a role as a platform for developing further research directions.

The analysis of LDA topic modeling for the last decade (2010–2020) will assist both scholars and practitioners in several ways. First, this study will inform scholars and practitioners regarding the trends and proportion of topics on sustainability and all three perspectives of marketing (micro-, meso-, and macro-marketing). This will inform researchers that the research range of sustainability and marketing is not limited to business-to-consumer relationship marketing [9,17,19,42,55], and highlighting how companies are environmentally friendly in the market [7,26,29,34,35,37,70]. Sustainability requires interdisciplinary study, and some argue that it is more interdisciplinary than other general scientific research. Previous studies have focused on sustainability as a part of marketing strategies, that is, aiming to positively influence consumers to have more sustainable consumption behavior or to make the market more sustainable. However, studies that consider sustainability as a marketing strategy must no longer consider sustainability as a marketing tool. It is evident in such studies that sustainability and marketing are investigated separately. Moreover, this study implies the impact of marketing on the inherent social desirability bias in the field of sustainability, which generally focuses on eco-friendly activities [26]. According to stakeholder theory, satisfying all stakeholders is important as it encompasses sustainability-related activities [44,47,49]. Hence, all the levels of marketing activities are required to be more than having the idea of being eco-friendly, and this study suggests specific directions of marketing in the sustainability field for both companies and scholars by showing the rise of topics for the past decade (i.e., environmental sustainability and the sustainability scenario of industry). Therefore, our results are valuable, as they show the relationship between a broad range of marketing perspectives and sustainability. However, as the perspective of marketing includes material processing for product consumption and developing relationships with companies [35,66,68,69], future research may broaden the research limitation based on the results of this study.

Second, this study distinguishes itself from other research by employing an unstructured machine-learning algorithm to reduce selection bias in identifying research topics. Moreover, to identify hot and cold topics, it is important that future research is conducted to establish a clearer understanding of research topic popularity rates. However, topic-modeling methods do not automatically provide valid outcomes. Text mining must be based on previous research because the algorithms only have a supporting role. Authors must strive to make valid and logical decisions, which range from selecting appropriate words for algorithms to interpreting and labeling topics. Our results present the frameworks analyzed to summarize the literature on sustainability and marketing. Therefore, analyzing the research trends of sustainability and marketing, in addition to grasping the topography, has important implications, which would support future research.

This study does not include the major analysis of certain topics that were considered in certain years, because the purpose of research trend investigation is to identify the hot and cold topics, irrespective of the detailed analysis on other topics that are statistically insignificant. However, certain topics that have been researched more than others in certain years must be justified. Hence, further research can be conducted to obtain a more detailed or holistic view of these topics by considering other strong marketing-related keywords, such as “supply chain,” “distribution,” and “product.” Studies that link marketing and sustainability fields during the last decade have been considered; however, they still represent only a small proportion of the literature. In the future, the countries, and disciplines that conduct the most or least research on sustainability and marketing should be investigated. How marketing scholars have settled into the sustainability category could also be an interesting direction for future research. Given the importance of these two trends and the need for more efficient strategies to implement sustainability, more robust and detailed research on sustainability and marketing must be performed in the future.

## Author contribution statement

Yeo Jin Jung: Conceived and designed the experiments; Analyzed and interpreted the data; Contributed reagents, materials tools or data; Wrote the paper.

Youngmin Kim: Conceived and designed the experiments; Performed the experiments; Analyzed and interpreted the data; Contributed reagents, materials tools or data.

## Funding statement

This work was supported by the Ministry of Education of the Republic of Korea and the National Research Foundation of Korea [NRF–2019S1A5B5A07107323].

## Data availability statement

Data included in article/supp. material/referenced in article.

## Declaration of interest’s statement

The authors declare no conflict of interest.
