# Supplementary material for: Research trends of sustainability and marketing research, 2010–2020: Topic modeling analysis
Source: Heliyon. 2023 Mar 5;9(3):e14208. doi: 10.1016/j.heliyon.2023.e14208 (PMC10025026; doi:10.1016/j.heliyon.2023.e14208)
Supplement: Multimedia component 1 [file mmc1.docx]

**Appendix.** Topic proportion over time (2010 – 2020)

|  | 2010 | 2011 | 2012 | 2013 | 2014 | 2015 | 2016 | 2017 | 2018 | 2019 | 2020 |
| --- | --- | --- | --- | --- | --- | --- | --- | --- | --- | --- | --- |
| (T1) | 0% | 5.0% | 11.3% | 3.3% | 0.9% | 2.8% | 3.4% | 3.4% | 4.7% | 1.8% | 2.5% |
| (T2) | 3.9% | 2.5% | 3.8% | 2.2% | 5.2% | 0.6% | 6.9% | 4.5% | 3.3% | 4.4% | 1.5% |
| (T3) | 7.7% | 2.5% | 3.8% | 5.6% | 7.0% | 8.1% | **14.1%** | 12.3% | 11.8% | 13.3% | 14.1% |
| (T4) | 3.8% | 2.5% | 0% | 4.4% | 8.7% | 2.9% | 4.6% | 6.0% | 4.4% | 4.2% | 7.0% |
| (T5) | 11.5% | 2.5% | 3.8% | 5.6% | 4.4% | 6.4% | 5.3% | 4.9% | 5.9% | 3.1% | 1.5% |
| (T6) | 0% | **15%** | 3.8% | 6.7% | 6.1% | 5.2% | 1.5% | 4.1% | 5.3% | 4.7% | 7.3% |
| (T7) | **23.1%** | 2.5% | 0% | 7.8% | 3.5% | 3.5% | 3.4% | 3.0% | 4.7% | 2.3% | 1.8% |
| (T8) | 3.9% | 10% | 7.6% | 11.1% | **16.5%** | **19.1%** | 11.8% | 9.7% | 16.6% | 14.6% | 14.3% |
| (T9) | 7.7% | 2.5% | 3.8% | 4.4% | 1.7% | 3.5% | 4.6% | 5.6% | 4.4% | 6.0% | 5.5% |
| (T10) | 7.7% | **15%** | 11.3% | 5.6% | 11.3% | 8.7% | 5.0% | 6.7% | 4.4% | 7.8% | 7.3% |
| (T11) | 11.5% | 12.5% | 9.4% | 3.3% | 8.7% | 8.1% | 6.9% | 6.7% | 2.7% | 4.7% | 5.5% |
| (T12) | 3.9% | 12.5% | **22.6%** | 11.1% | 13.9% | 11.6% | 13.4% | **20.2%** | **17.8%** | **20.3%** | **16.3%** |
| (T13) | 7.7% | 12.5% | 5.7% | **22.2%** | 8.7% | 12.1% | 13.7% | 7.5% | 9.5% | 7.6% | 9.3% |
| (T14) | 7.7% | 2.5% | 13.2% | 6.7% | 3.5% | 7.5% | 5.3% | 5.6% | 4.4% | 5.2% | 6.0% |
